# One step closer to a transmissible vaccine for rabies virus

**DOI:** 10.1371/journal.pbio.3001607

**Published:** 2022-04-20

**Authors:** Scott L. Nuismer

**Affiliations:** Department of Biological Sciences, University of Idaho, Moscow, Idaho, United States of America

## Abstract

This Primer explores a recent study in PLOS Biology showing that a betaherpesvirus circulating in the vampire bat could serve as an effective vector for a transmissible vaccine capable of reducing the risk of rabies virus spillover in Peru.

## Teaser

A recent study in *PLOS Biology* shows that a betaherpesvirus circulating with the vampire bat, *Desmodus rotundus*, could serve as an effective vector for a transmissible vaccine capable of reducing the risk of rabies virus spillover in Peru.

Transmissible vaccines offer a powerful approach for reducing the prevalence of infectious disease within populations of wild animals. By transmitting autonomously from one animal to the next, these vaccines multiply the impacts of direct vaccination and make establishing and maintaining herd immunity in wild animal populations more financially and logistically feasible. Although there are many routes to the development of a transmissible vaccine, the safest and most elegant inserts a gene from the target pathogen into the genome of a benign and naturally circulating vector virus. By choosing a pathogen gene that is recognized by the immune system of the animal reservoir, this procedure endows the vector virus with the ability to stimulate immunity to the target pathogen. If carrying the foreign gene is not too burdensome, and immunity to the viral vector not already firmly established, the resulting vaccine will transmit among reservoir animals, immunizing them as it goes [1]. Despite the outward simplicity of this approach, it has yet to be achieved for any pathogen that regularly spills over into the human population. One roadblock has been the lack of a suitable viral vector.

A new study published in *PLOS Biology* by Griffiths and colleagues pushes us closer to a transmissible vaccine for rabies virus by identifying a promising viral vector [2]. Their work focuses on *Desmodus rotundus* betaherpesvirus (DrBHV), a double-stranded DNA virus circulating within Peruvian populations of vampire bats. Betaherpesviruses like DrBHV have long been considered to be candidates for transmissible vaccine development because they are thought to be species specific, largely benign in healthy host animals, and capable of superinfection [3–5]. Until now, however, we had little understanding of betaherpesvirus epidemiology within natural populations of animals that serve as reservoirs for important human pathogens.

**Fig 1:**
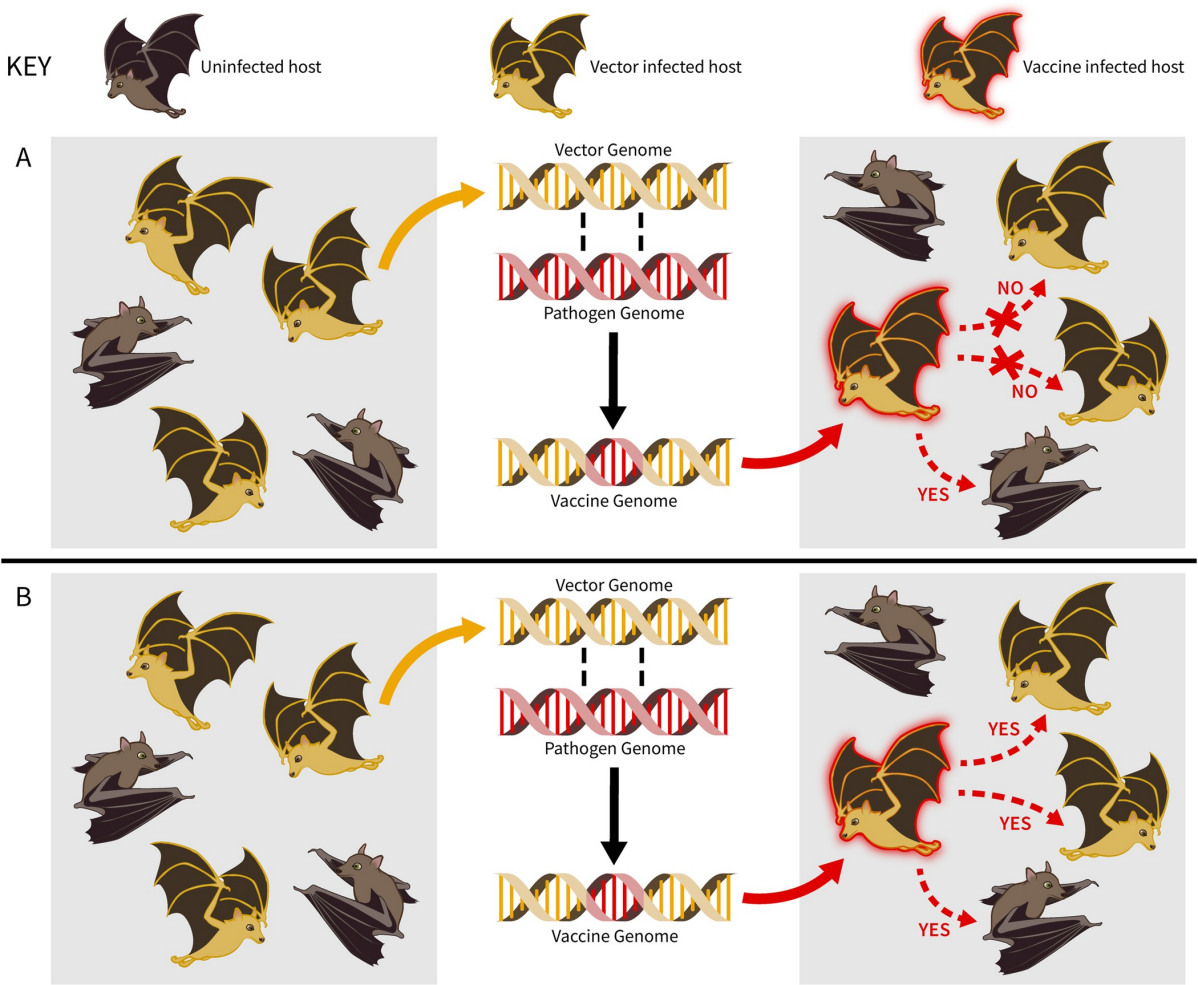
A recombinant vector transmissible vaccine is constructed by isolating the vector virus from a population of the animal reservoir in which it naturally circulates (left-hand panels). The vector virus is brought back to the lab where it is engineered to carry a gene of the pathogen that stimulates an immunizing response within the reservoir (center panels). The newly created transmissible vaccine is then released back into the population from which the vector virus was isolated (right-hand panels). If prior infection of reservoir animals by the naturally circulating vector virus blocks reinfection by the vaccine, the gains made by vaccine transmission will be minimal (top row, A). In contrast, if the vaccine can reinfect animals previously infected with the naturally circulating vector virus, the gains made by vaccine transmission can be substantial (bottom row, B).

The key advance made by Griffiths and colleagues is the use of genomic sequencing to decompose gross patterns of DrBHV infection into infections by genetically distinct strains. This decomposition reveals that DrBHV can infect bats previously infected by other strains of DrBHV, suggesting that preexisting immunity may not impede a transmissible vaccine using DrBHV as a vector (Fig 1). Two lines of evidence support this conclusion. First, individual bats are commonly infected by multiple strains of DrBHV. Second, by capturing bats the team had captured previously, Griffiths and colleagues inferred that some had become superinfected by novel strains of DrBHV. Although these results convincingly show that superinfection is possible, follow-up work will be needed to quantify how the likelihood of superinfection depends on the genetic distance between the initially infecting strain and those that follow.

By decomposing infection by DrBHV into infections by individual strains, Griffiths and colleagues also show that the prevalence of individual strains varies across bat populations. Encouragingly, some strains reached local prevalences that would be sufficient for rabies elimination if they were acting as transmissible vaccines. Other strains were generally rare and had prevalences insufficient for rabies elimination. This result raises a vexing question: How do we select which vector strain to use as a backbone for a transmissible vaccine? Griffiths and colleagues use a sophisticated combination of analyses to argue for a general dynamic where initially rare strains increase in frequency, spread broadly, and then are rapidly replaced. This argues for a strategy focused on selecting rare strains, as does the potential to avoid preexisting strain-specific immunity. There are many reasons a strain could be rare; however, most of which would adversely impact its ability to serve as a vector. For instance, rare strains could be poorly adapted to the local host population or have fallen from earlier dominance as strain specific immunity increased. Future work will need to identify methods for a priori screening of vector strains, perhaps using mathematical models that can distinguish between strains that are likely to increase in prevalence over the short term and those that will most likely decrease in prevalence.

Despite the exciting progress the work of Griffiths and colleagues represents, significant obstacles remain to be overcome. Most obviously, DrBHV would need to be engineered to immunize vampire bats against rabies without interfering with its ability to replicate and transmit. This is a complex challenge that contributed to the failure of efforts to develop transmissible immunocontraceptive vaccines for invasive wildlife [4]. The inevitability of vaccine evolution poses an additional array of challenges. For instance, adaptation to cell culture during vaccine development may lead to attenuation and a failure of the vaccine to transmit effectively [4]. Alternatively, mutations that silence or eliminate the foreign gene required for DrBHV to function as a vaccine may be selectively favored and sweep through the vaccine population [1]. Although harmless, the evolutionary loss of transgene function could disable the vaccine before meaningful levels of immunity can be established in the reservoir population. Evolutionary loss of transgene function, even if slow, could also inhibit the spread of the vaccine by creating competing lineages freed from the burden of carrying the foreign gene. Although surmounting these remaining challenges will require significant reseources and research, the work of Griffiths and colleagues has moved us closer to the point where these challenges can begin to be solved.
